# ADHD Symptoms in Adults and Time Perspectives – Findings From a Czech National Sample

**DOI:** 10.3389/fpsyg.2020.00950

**Published:** 2020-05-28

**Authors:** Simon Weissenberger, Filip Děchtěrenko, Martina Klicperova-Baker, Martina Vňuková, Phillip Zimbardo, Jiří Raboch, Martin Anders, Ellen Braaten, Radek Ptáček

**Affiliations:** ^1^First Faculty of Medicine, Department of Psychiatry, Charles University, Prague, Czechia; ^2^Department of Psychology, University of New York in Prague, Prague, Czechia; ^3^Institute of Psychology, Czech Academy of Sciences, Prague, Czechia; ^4^Department of Psychology, Stanford University, Stanford, CA, United States; ^5^Second Faculty of Medicine, Department of Child Psychiatry, Charles University, Prague, Czechia

**Keywords:** ADHD, Zimbardo, time, addiction, adults

## Abstract

**Introduction:**

Attention deficit hyperactivity disorder (ADHD) is one of the most common neurodevelopmental disorders, affecting individuals in all stages of their lives and leading to a variety of negative quality of life outcomes. The disorder is associated with marked differences related to time perception and time perspectives, and this area of research is currently becoming more prominent and gaining ground in showing new aspects of ADHD that were considered secondary (i.e., time perception differences, affective differences). In this study, we looked at ADHD symptoms in adults, correlated lifestyles, and time perspectives as defined by the Zimbardo Time Perspective Inventory (ZTPI). The ZTPI is a useful standardized scale to measure one’s time perspective anchoring in the categories of past positive, past negative, present fatalistic, present hedonistic, and future oriented. This is the first study on adult ADHD and time perspectives conducted in the Czechia.

**Methodology:**

A national representative sample of Czech adults aged 18–65 was recruited by the STEM/MARK Agency. The individuals were assessed for ADHD symptoms with the Adult ADHD Self-Report Scale (ASRS v.1.1). Furthermore, a demographic and lifestyle questionnaire was administered along with the ZTPI to assess time perspectives. Statistical calculations were conducted to find correlations between ADHD symptoms as assessed by the ASRS and the various categories of the ZTPI.

**Results:**

ADHD symptoms were found to be positively correlated with the present hedonistic perspective along with the past negative perspective. Gender was a strong factor in both ADHD symptoms, with males being more likely to show symptoms and to have a present hedonistic perspective. In females, the past negative perspective was most prominent. Education and age were negatively correlated with ADHD symptomatology and the present hedonistic perspective also decreased with age unlike the past negative perspective. Other time perspectives such as future orientation was seen in individuals with lower ADHD symptoms and higher levels of educational achievement.

**Conclusion:**

Researching ADHD symptoms and their connection to time perspectives can increase knowledge of both the disorder and how time perspectives tie into it. We wish to also raise awareness of the possible utility of the ZTPI scale when working with individuals with ADHD.

## Introduction

Attention deficit hyperactivity disorder (ADHD) is currently the most common neurodevelopmental disorder in the world. It is estimated to affect between 5 and 7% of the world population in terms of diagnosis, and it impacts individuals in all stages of life (Polanczyk et al., 2007; Filipe and Bergey, 2018). The disorder is associated with a variety of symptoms that include hyperactivity, inattention, and impulsivity, among others. It is subdivided into ADHD predominantly inattentive (ADHD I), predominantly hyperactive/impulsive (ADHD HI), or combined (ADHD C). To be diagnosed, one must display at least five symptoms from the hyperactive category and/or the inattentive; furthermore, the symptoms must occur before age 12 (APA, 2013). The disorder is most commonly diagnosed in males and is associated with a wide variety of negative health outcomes. These range from higher risks of cardiovascular disease from poor dieting, higher rates of smoking, to higher rates of mortality from accidents (Nigg, 2013). In recent years, there has been a growing interest in investigating the impact and connection between altered time perception and ADHD in both children and adults. Time perception had long been considered a secondary aspect of the disorder but is now gaining more traction as being a possible core element of ADHD (Smith et al., 2002; Barkley, 2016). Part of our current research is also actively investigating the mechanisms and correlations between time perception and ADHD symptomatology. A more pragmatic approach we are taking is connecting ADHD to specific time perspective as laid out by the standardized scale known as the Zimbardo Time Perspective Inventory (ZTPI).

The ZTPI is a standardized scale devised by Zimbardo and Boyd in 1999, which aims to understand people’s time perspective in terms of cognitive and affective attention toward the past, present, or future. The categories in the questionnaire are the past positive, past negative, present fatalistic, present hedonistic, and future orientation. Individuals who have had negative or traumatic experiences in the past tend to score higher in the past negative; others base their current experiences on past positive events whether real or imagined. The present fatalistic category describes people who feel that their life is ruled by outside forces and have an external locus of control. The present hedonist is ruled by pleasure-seeking activities and enjoying the moment, and the future-oriented individual tends to be making plans for the future and effectively is able to postpone gratification (Zimbardo and Boyd, 1999). In 2016, we had conducted a literature review looking at associated lifestyles and comorbidities seen in those with ADHD and found a significant amount of literature related to poor diet, addictive behaviors such as drug addiction and video game addiction, along with delinquency and other issues. This led us to hypothesize that individuals with ADHD symptoms are likely to fall in the present hedonistic category of the ZTPI. Furthermore, we thought that research on the topic of expanding the use of the ZTPI could be helpful in the clinical setting in terms of giving an additional tool for treatment and management of the disorder (Weissenberger et al., 2016). We later also conducted the first study in the Czechia looking at adult ADHD symptomatology and correlating certain lifestyles. Although most of the findings we found were similar to previous data, we found some paradoxical findings such as negative correlations with nicotine use and ADHD symptoms and also high awareness of individuals with ADHD of their unhealthy lifestyle habits (Simon Weissenberger et al., 2018). In the present study, we follow up with Czech adults and, besides lifestyles, we also look at previous treatment and make correlations with the ZTPI to see whether our hypothesis was correct or not.

## Methodology

### Participants

A sample of adults aged 18–65 years from the Czechia was recruited by the professional polling and statistics agency STEM/MARK, a.s. The subjects were randomly chosen from a Czech National Panel, and the questioning was done through a computer-assisted web interviewing method. The sample of Czech adults was *N* = 1,518 consisting of 766 males and 752 females.

### Exclusionary Criteria

The following were all included as the exclusion criteria for the sample: the presence of a severe neuropsychiatric disorder such as intellectual disability, schizophrenia, psychosis, dementia, substance dependence or behavioral addiction, or neurodegenerative diseases. Other exclusionary aspects were severe somatic disorders with a direct effect on cognitive function such as cardiovascular, cerebrovascular, and/or endocrinological diseases as well as the use of drugs that affect cognitive functioning.

### Measures

The CAWI method of online questioning was used to administer the questionnaires. The program was deemed the most effective for our cross-sectional study. Informed consent in the study was ensured and respondents were informed about the possibility to not answer questions and their right to withdraw from the study altogether. The study was approved by the Ethical Committee of the First Faculty of Medicine of Charles University in Prague. The participants were administered with the ASRS to determine ADHD symptoms and severity. The scale is intended for adults and divides scores from 0 to 6 with 0 representing no ADHD symptoms to 6 being very severe symptoms. Score ranges of 0–3 usually imply that the person does not have ADHD; score ranges of 4–6 require clinicians to investigate as the person is very likely to have ADHD. The questionnaire has been validated and found a reliable tool for assessing ADHD symptoms in adults (Adler et al., 2006). Alongside the ASRS test, the ZTPI was also administered; this standardized questionnaire places people into categories related to their cognitive time anchoring and into time perspectives; these include the past positive, past negative, present fatalistic, present hedonistic, and future perspectives (Zimbardo and Boyd, 1999). We further extended the Likert scale with the “I do not know” option, which allowed us to extend the analysis for patterns of incomplete values. Furthermore, we developed a demographic and lifestyle questionnaire that looked at basic information such as age, sex, and educational level along with family history of ADHD, any previous diagnosis and treatment, comorbid conditions such as insomnia, and other psychiatric conditions. We also asked about previous problems in school settings and legal troubles related to everyday activities such as driving a vehicle. We then proceeded with a variety of lifestyle questions related to issues such as diet, amount of physical activity performed weekly, drugs of abuse, and medication, among others. Correlations were later made in terms of ADHD symptom severity, age, gender, associated lifestyles, and associated time perspectives.

### Data Analysis

The data were analyzed with the statistical software R (R Core Team, 2019). The analysis was performed as follows: We focused on three demographic variables (sex, education, and age). For the analysis of the ZTPI scales without including the ASRS questionnaire, we first tested the differences using multivariate analysis of variance (MANOVA) between sex and education followed by tests for each scale (*t*-tests for sex and a one-way ANOVA for education).

In the case of the ASRS questionnaire, we used two approaches. First, we computed the correlations of the ASRS score (range of 0–6) with the ZTPI scale categories. Second, we divided the participants into low (0–3 score) and high (4–6 score) ADHD symptoms and tested the relationship between ZTPI categories and demographic variables. This approach led to two-way ANOVA (in the case of sex and education) and one-way ANCOVA (in the case of age) (R Core Team, 2019).

## Results

From the total amount of participants, 119 (or 7.8%) scored as high on ADHD symptoms. Similar percentages were obtained for both males (8.2%) and females (7.45%). Furthermore, there was a statistically significant reverse relationship between age and ASRS scores. Similarly, when looking at people’s educational level, the higher the levels achieved (i.e., university education), the lower the ADHD symptoms [for more details, refer to Vnukova et al. (2020), in preparation].

The statistical analysis and calculations for the ZTPI scales in the Czech national sample we assessed revealed a similar pattern to a previous study conducted in the Czechia in 2013 (Lukavská et al., 2011). The full data set, including means for subgroups defined by sex and education categories, is shown in Table 1.

**TABLE 1:** Mean (SD) for ZTPI scales including reliability mean (SD) for each individual scale.

ZTPI scale	Overall	Sex		Education				
		Female	Male	Elementary	College without diploma	College with diploma	University	Cronbach’s α [95% CI]
Past Negative	3.09 (0.75)	3.14 (0.72)	3.04 (0.77)	3.34 (0.71)	3.25 (0.74)	3.03 (0.72)	2.83 (0.73)	0.86 [0.85, 0.87]
Past Positive	3.39 (0.56)	3.41 (0.57)	3.37 (0.53)	3.32 (0.61)	3.34 (0.56)	3.44 (0.55)	3.42 (0.51)	0.66 [0.64, 0.69]
Present Fatalism	2.80 (0.69)	2.84 (0.67)	2.76 (0.71)	2.98 (0.69)	2.99 (0.68)	2.75 (0.67)	2.49 (0.61)	0.81 [0.80, 0.82]
Present Hedonism	3.13 (0.56)	3.14 (0.54)	3.13 (0.59)	3.38 (0.51)	3.22 (0.60)	3.12 (0.54)	2.92 (0.48)	0.83 [0.82, 0.84]
Future	3.46 (0.46)	3.51 (0.44)	3.42 (0.47)	3.32 (0.44)	3.39 (0.45)	3.49 (0.47)	3.58 (0.44)	0.70 [0.67, 0.72]

Full descriptive data including means for subgroups defined by sex and education categories are shown in Table 1.

Past Negative, Present Fatalism, and Present Hedonism showed high reliability (Cronbach’s α ≥ 0.81); Positive Past and Future showed reliability just above acceptance (Cronbach’s α ≥ 0.66). Multivariate analysis variances between males and females revealed significant differences (Pillai’s trace = 0.12, *p* < 0.001). Differences in each scale revealed significant differences between males and females in the Past Negative category [*t*(1,366) = 2.61, *p* = 0.009], Present Fatalism [*t*(1,319) = 2.12, *p* = 0.034], and Future [*t*(1,391) = 3.80, *p* < 0.001].

Multivariate analysis of variance showed significant differences between achieved education (Pillai’s trace = 0.12, *p* < 0.001). Differences between achieved education was significant for each scale (*p’*s ≤ 0.027). In general, Past Positive and Future increased with education, while Past Negative, Present Fatalism, and Present Hedonism decreased with education as described in Table 1.

The analysis of correlation forage and ZTPI categories revealed that both Past Negative and Present Hedonism decreased with age (past negative: *r* = −0.13, present hedonism: *r* = −0.22), while the other three categories did not change with age. The full correlation table between categories is shown in Table 2.

**TABLE 2:** Correlation matrix for ZTPI scales, age, and ASRS score.

	Age	ASRS score	Negative past	Positive past	Fatalistic present	Hedonistic present
**Age**						
ASRS score [0–6]	−0.21***					
Past Negative	−0.13***	0.16***				
Past Positive	0.02	−0.08**	–0.04			
Present Fatalism	–0.04	0.12***	0.60***	0.01		
Present Hedonism	−0.22***	0.17***	0.35***	0.18***	0.52***	
Future	0.02	−0.16***	0.04	0.22***	−0.19***	−0.13***

** *p* < 0.01, *** *p* < 0.001.

**FIGURE 1:**
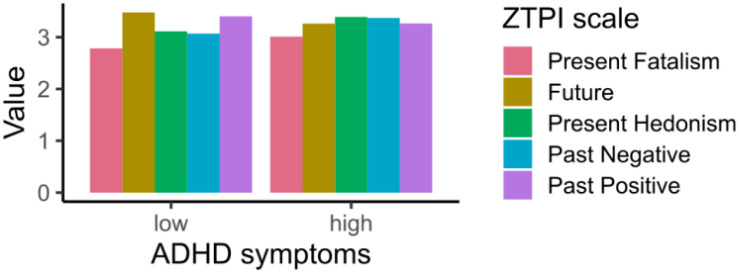
Profile graph for participants with low and high ADHD symptoms.

**FIGURE 2:**
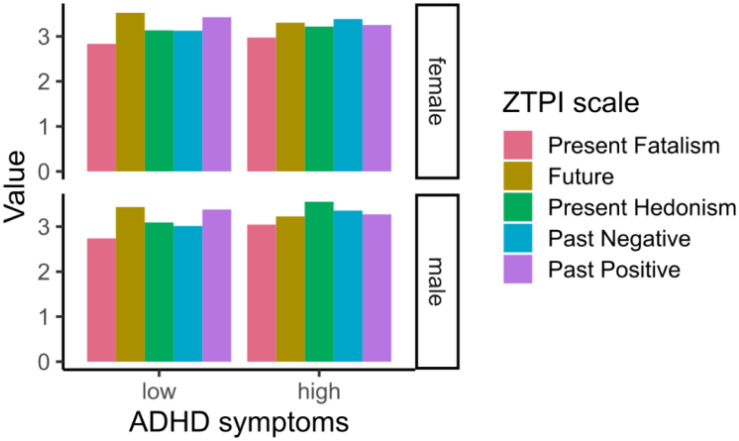
Profile graph for participants with low and high ADHD symptoms broken down by sex.

The correlation between ZTPI categories and ASRS scores is shown in Table 2. In general, all scales showed a correlation with the ASRS scores, while Past Positive and Future decreased with higher ASRS score (Past Positive: *r* = ***−***0.08, Future: *r* = ***−***0.16); the remaining three increased with ASRS score (Past Negative: *r* = 0.16, Present Fatalism: *r* = 0.12, Present Hedonism: *r* = 0.17). In the following text, we will focus on the differences between the ZTPI categories when we divide participants into high and low ADHD symptom groups. From the profile graphs in Figure 1, which show the average values for each category divided by ADHD symptomatology, we can see that Present Hedonism is the most dominant time perspective in participants with high ADHD symptoms followed by the Past Negative category. The differences between participants with high/low ADHD symptoms are significant for each scale (all *p’*s ≤ 0.044). When the participant’s sex is added as an additional factor, the present hedonistic time perspective is predominant only in males with high ADHD symptoms, while in females, the Past Negative is the most predominant time perspective (Figure 2). Again, the differences between high and low ADHD symptomatology groups remained significant (all *p’*s ≤ 0.044), and the differences between males and females showed the same significance as described before. The interaction of ADHD symptoms and sex was significant only for the Present Hedonism category (*p* = 0.001).

**FIGURE 3:**
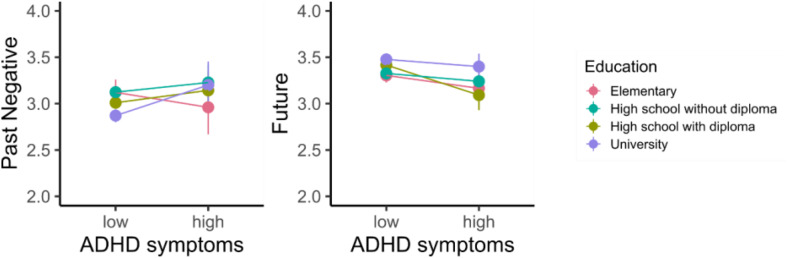
Interaction between ADHD symptoms and education for negative past and future. Vertical lines denote 95% bootstrapped confidence intervals.

By testing the differences in participants with high/low ADHD symptomatology and achieved education, the differences between low/high ADHD subgroups remained significant (*p’*s ≤ 0.016), and also the differences between education were significant in similar pattern as without ADHD symptomatology. Moreover, the interaction between ADHD symptoms and education was significant for Past Negative and Future as shown in Figure 3.

By adding education into the model, the differences between ADHD groups are still significant. Moreover, the groups differ in education in every scale, and additionally, there is an interaction with the Future time scale and the Past Negative category as visualized in the following figure. The decrease in Future time perspective was greatest for those with a completed high school education. In the case of Past Negative, for participants with elementary education, the negative scale decreased with higher ADHD symptoms, while for higher education, it increased.

**FIGURE 4:**
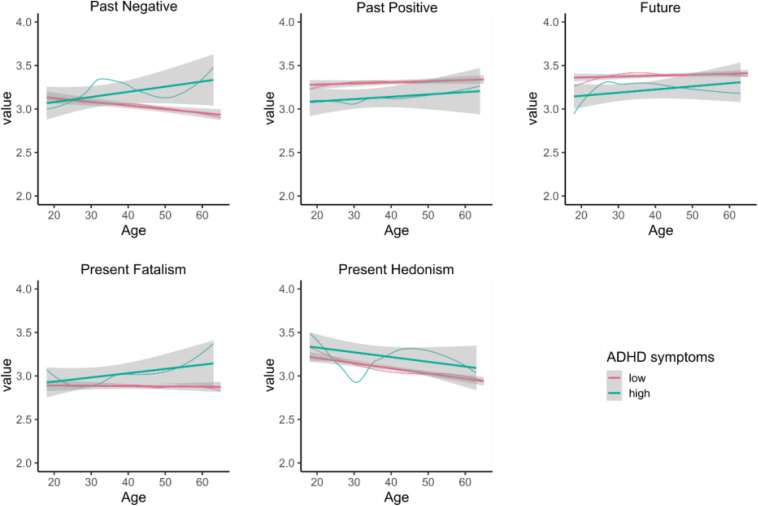
Relationship between ZTPI scale score and age for participants with low/high ADHD symptoms. For each group, bold regression line with 95% confidence band is shown as well as local regression (loess) estimate showing subtle patterns (thin curve).

Finally, we explored the relationship between age and ZTPI categories when we add ADHD symptomatology into the model. The differences between ADHD symptomatology subgroups remained significant (*p’*s ≤ 0.018). The relationship between age and Past Negative decreased for Past Negative and Present Hedonism. In Figure 4, we show both a linear regression showing the general trend as well as the local polynomial regression (locally estimated scatterplot smoothing: loess), which can capture subtle trends in data.

Because we analyzed differences between participants with low/high ADHD symptoms using only one variable, we created a single large model with all the factors added at once (ADHD symptomatology, sex, education, and age) including all the interactions (this would lead to a four-way ANOVA). With the exception of one three-way interaction between ADHD symptomatology, sex, and education in the Past Positive category, all high-level interactions were non-significant, while lower level interactions showed the same results as a simpler analysis. The interaction in the case of Past Positive was slightly below significance [*F*(3,1367) = 2.73, *p* = 0.043] and becomes non-significant after correcting for multiple comparisons; thus, we did not explore this relationship further.

Additionally, we also analyzed percentages of responses of “I do not know” in the ZTPI. Table 3 shows that with the exception of Present Fatalism, participants with higher ADHD symptomatology showed a higher number of “I do not know” responses.

**TABLE 3:** Percentages of “I don’t know” responses for each ZTPI scale.

	ADHD symptoms	
ZTPI scale	Low (%)	High (%)
Past Negative	9.51	14.29
Past Positive	7.65	10.08
Present Fatalism	13.01	12.61
Present Hedonism	11.87	15.13
Future	8.01	10.92

## Discussion

The aim of this study was to investigate the connection between ADHD symptoms in adults and time perspectives as measured by the ASRS and the ZTPI, respectively. We believe that time perspective can be a useful tool that can have future use in the clinical and psychological realm. In the case of ADHD, the association to an altered sense of time or time perception is well known, but a more concrete and applicable psychometric tool such as the ZTPI could be quite helpful in both the diagnosis stage as well as in the treatment of the disorder. Our study partially confirmed a previous hypothesis article that we wrote in 2016 where we stated that the most likely time perspective from the ZTPI that individuals with ADHD would be anchored in is the present hedonistic category (Weissenberger et al., 2016). This was found to be true in the males from our study, who also happen to be the most likely to be diagnosed with ADHD in general. It is also known that externalizing behaviors generally associated with impulsivity and what we may understand as a hedonistic outlook were also found in males throughout their lifespan. Furthermore, in women, internalizing disorders such as depression and anxiety are more common and widespread (Yoshimasu et al., 2018). Interestingly, in our study, women scored the highest in the past negative category in the ZTPI. This category is associated with viewing the present in light of negative events that have occurred in the past. This is associated with a wide range of psychological and cognitive phenomena, from the experience of traumatic events to a heightened sensitivity to physical pain (Zimbardo and Boyd, 1999; Gács et al., 2020). It should be noted that just as Zimbardo had previously looked at issues such as posttraumatic stress in soldiers in relation to the past negative perspective, other studies found higher rates of trauma in young girls with ADHD (Zimbardo and Boyd, 1999; Elton et al., 2014). Another factor that confirmed previous literature on the topic was the association of a future time perspective with better educational outcomes; this was confirmed even among individuals who had strong ADHD symptoms but had a university education. Although it was not looked at for the purpose of this study, those with ADHD who had gained a university degree may have been medicated. This would confirm that ADHD drugs can have a normalizing effect on ADHD symptoms, and that these are. Another research had previously found that individuals with ADHD who were on some kind of ADHD medication were more likely to be future-oriented according to the ZTPI (Carelli and Wiberg, 2012). The connection that we saw between aging and higher rates of past negative was also connected in previous studies related to depression (Åström et al., 2019).

This study had a few limitations including its cross-sectional design and some of the exclusion criteria for the participants. There will be a follow-up to this study but with only 899 of the total 1,518 participants who have agreed to take part exploring other aspects of time perception such as time estimation and discrimination and its connection to ADHD symptoms. The exclusionary criteria included some of the comorbid conditions often seen in ADHD such as other serious mental health conditions and serious addictions. This was necessary to get a more accurate analysis of ADHD symptoms and to not misrepresent the data. Another possible limitation was that unlike the study conducted by Carelli and Wilber in 2012, we relied on the original ZTPI, which has a future orientation but does not differentiate among Future Positive and Future Negative. Also, unlike other studies, we focused on symptomatology in the general adult population rather than focusing on ADHD diagnosed individuals. This is because there is still a strong bias in seeing ADHD as a childhood condition among Czech mental health practitioners. Many adults with ADHD symptoms in the Czechia may never be diagnosed with the condition and may suffer because of this bias. This was one of the main reasons for our previous study on the widespread of ADHD symptoms in Czech adults and their associated lifestyles in 2018; please see Weissenberger et al. (2018) for more on that. Other studies have looked at ADHD symptoms, time perspectives, along with other issues such as addictive behaviors. For example, Settanni et al. (2018) looked at the correlation between ADHD symptoms and addictive Facebook use along with time perspectives. Unlike our study, they focused primarily on adolescents and interestingly found the addictive behavior correlated with ADHD symptoms and a Present Fatalistic or Past Negative perspective. There are not so many studies currently looking at ADHD and time perspectives, but we believe that investigating this aspect of the condition can be quite useful for therapeutic purposes and as an additional tool for the diagnosis of the disorder, especially in adults.

## Conclusion

There is a clear connection between ADHD symptoms in adults and a present hedonistic time perspective in males and a past negative time perspective in females. Present hedonism was negatively correlated with advanced age, while the past negative perspective had a positive correlation. Individuals with higher levels of education were also more likely to have a future time perspective. In terms of furthering research and improving treatment outcomes for ADHD, we strongly recommend the inclusion of time perspectives when working with individuals with ADHD. This can help foster new therapeutic methods and also raise better awareness of the disorder.

## Data Availability Statement

All datasets generated for this study are included in the article/supplementary material.

## Ethics Statement

The studies involving human participants were reviewed and approved by First Faculty of Medicine, Department of Psychiatry, Charles University Ethics Committee. The patients/participants provided their written informed consent to participate in this study.

## Author Contributions

SW contributed to ideating the research, literature review, and analysis of data. FD contributed to the statistical analysis and analysis of data. MK-B contributed to the literature review and data analysis. MV involved in data analysis and manuscript revisions. PZ, JR, MA, and EB contributed as research grants. RP involved in supervising the research and analysis of data.

## Conflict of Interest

The authors declare that the research was conducted in the absence of any commercial or financial relationships that could be construed as a potential conflict of interest.
